# An overview on H5N1 virus: Recent outbreaks and challenges

**DOI:** 10.6026/973206300210544

**Published:** 2025-03-31

**Authors:** Atul Rukadikar, Shruti Dalpatbhai Kalola, Srushti J. Bhoraniya, Komal Ravi Thaker, Charushila Rukadikar

**Affiliations:** 1 Department Microbiology, All India Institute of Medical Sciences, Gorakhpur, Uttar Pradesh, India; 2 Department of Health, CHC Dhrol-Jamnagar, Gujarat, India; 3 Department of Health, PHC Lajai-Morbi, Gujarat, India; 4 Department of Anesthesiology, Medical Science and Research, Dharmasinh Desai University, Nadiad, Gujarat, India; 5 Department Physiology, All India Institute of Medical Sciences, Gorakhpur, Uttar Pradesh, India

**Keywords:** H5N1 virus, case fatality rate, fatalities, western pacific region, significance

## Abstract

Avian influenza - A (H5N1) is a type of virus that causes highly infectious, severe respiratory influenza disease in birds. It also infects mammals rising concerns about its re-assortment and increased virulence. New strains with mutations have been making the virus more pathogenic. The more likely source for disease outbreak is from dairy cows or chickens that are infected. This presents a considerable source of economic detriment for both agricultural producers and the food industry. Hence, a concise overview on H5N1 to enhance the understanding of its infection for adequate disease combat and management is relevant.

## Background:

According to a study, avian influenza virus s pose substantial challenges to global public health systems attributed to their extensive circulation and notable mortality rates [1]. According to studies, within the influenza A genus, they have a genome made up of eight segments that code for at least 11 different proteins, out of which hemagglutinin (HA) and neuraminidase (NA) in avian species are categorized into 16 and 9 subtypes [2, 3]. There are different types of avian influenza virus because these two proteins are split into different groups. This is because their genes are different [2]. Studies have shown that "they are classified into two distinct categories according to their pathogenicity index (IVPI) test: highly pathogenic avian influenza viruses and low pathogenic avian influenza viruses [4, 5]. In recent years, various regions across the world have been affected by an infection caused by the HPAI H5N1 virus, which has raised concerns among authorities in charge of global health [6, 7]. The United States of America and other nations across the globe have been affected by this infection, which has become the virus that has caused the most widespread outbreak among wild birds [8]. This outbreak, which spread across more than 28 European countries revealed a significant number of this virus across hosts (wild birds, poultry and domestic birds) [8]. In another study, the possibility of transmission between sea lions and pelicans has been brought to light, bringing to light the complex dynamics of viral transmission among these many species of animals [9]. Therefore, it is of interest to document an overview on H5N1 virus with recent outbreaks and its management challenges.

## Current outbreak in mammals:

In October of 2022, a mink farm in Spain provided the first convincing proof that H5N1 may transfer from one animal to another in a field environment [10]. According to past studies, during July to December 2023, a second, more significant H5N1 outbreak took place on 71 farms in Finland, which impacted American mink (6 farms), arctic foxes (64 farms) and raccoon dogs (5 farms) [11, 12]. According to studies, these adaptations included polymerase basic protein 2 alterations, such as PB2 (T271A) [13, 14]. Based on the close genetic similarity of the viruses found on several farms, it was hypothesized that animals spread them. The viruses were able to transfer effectively between ferrets that were in direct touch with one another, according to the findings of both experimental experiments [15, 16]. Consequently, it was posited that the disease was transmitted across farms by the movement of contaminated equipment, clothing, or the carcasses of infected mink fed to others [11].

## Pathogenesis in humans:

According to studies, this virus is caused by a complicated process that includes many things, but the main ones are viral replication, misregulation and increase of cytokines, chemokines macrophages and respiratory epithelial cells. It has been shown that immune factors that are out of balance, like TRAIL levels going up and CD8+ cells not killing as many cells, are linked to the disease getting worse and to the pathological features seen in human cases [17, 18 19-20. Apoptosis is a key pathogenic process that starts with direct viral replication and/or the production of cytokines and chemokines [18]. Furthermore, lymphopenia associated with influenza has been identified as an additional mechanism contributing to the pathogenesis of H5N1, especially in severe cases observed in humans [21]. On the other hand, another study showed, new mechanism which is characterized by the reduction of performing activity in cytotoxic T cells due to the influence of hemagglutinins that result in a reduction of cytotoxic activity, hindering the elimination of infected cells, such as antigen-presenting cells (APCs)harboring the virus [22]. According to a study, the incubation period of infection is brief, (around seven days or less) [23]. According to studies, individuals may develop headaches, muscular soreness, sore throats, runny noses and, less often, conjunctivitis or bleeding gums (Figure 1 - see PDF) [24, 23]. Studies also found that "lungs infected due to this virus commonly undergo diffuse alveolar destruction, bleeding, formation of hyaline membrane, lymphocytic infiltration and fibroblast presence [18, 23]. Another study has demonstrated that the virus can penetrate the CNS through the olfactory nerve, leading to severe meningo-encephalitis [25]. Hepatic problems from flu are not common, but they have been linked to higher levels of transaminase [23]. These consequences have led to the need for hospitalization. People who have been infected with HPAI H5N1 are more likely to die if they have high viral loads, low lymphocyte counts and high levels of inflammatory cytokines and chemokines [18].

## Risk for pandemic:

Transmission from mammals to mammals is a big part of epidemics involving minks, pinnipeds and dairy calves and the fact that infections stay in different types of mammals is very worrying [26]. Researchers have used ferret experiments to evaluate the possibility of transmission among mammals. Ferrets can contract infections and it has been demonstrated that these viruses can be transmitted through direct contact with naïve ferrets residing in the same enclosures, leading to the development of disease. But the virus can't get from infected ferrets to naïve ferrets that are separated from the infected animals by a perforated barrier when it is spread through aerosols [27]. According to a study, several genetic changes that can lead it to pandemic condition like (1) change of receptor from avian type to human type i.e. α2.3 to α2.6 linked sialic acid (2) increased HA thermostability and pH (3) Increased replication at temperature equivalent to human upper respinfluenza risk assessment toolory tract (4) change in expression of RNA transcipt by viral polymerase complex (5) change in PB2 protein (single amino acid) (E627K) lead to change in polymerase activity [27].

## Vaccine:

The food and drug administration has approved three H5N1 vaccines [28], but there are no H5N1 vaccines available right now and none of the medicines that have been approved so far contain clade 2.3.4.4b HA components. The vaccine can't be made available unless the food and drug administration either grants emergency use permission or opens an extended access process for an experimental H5 vaccine. Any H5 vaccine would need a 2-dose adjuvant vaccine due to the vaccine's low immunogenicity. The development and evaluation of candidate influenza vaccine viruses for novel influenza strains is a typical aspect of pandemic planning. There are currently active viruses that might cause a pandemic [29]. The Center of disease control and prevention and food and drug administration /Center for Biologics Evaluation and Research have produced H5 candidate vaccine viruses against the clade 2.3.4.4b virus. Manufacturers now have access to these viruses and can potentially use them to develop vaccines [29]. The Center of disease control and prevention Influenza Risk Assessment Tool aids in the selection of viruses for vaccine candidate development [30].

## Recent report:

World health organization in December 13, 2024 released recent update of new cases (C) and death (D) starting from January 1, 2023 ending to November 1 , 2024) where they suggested that, 939 cases of human infection with H5N1 virus were reported from 24 countries. Of these 939 cases, 464 were fatal (CFR of 49%) [31]. Table 1 shows the recent updated world health organization report.

## Guideline for prevention [32]:

## Generic protocol:

[1] Standard precaution like hand hygiene, glove and personal protective equipment (personal protective equipment) kit

[2] Prevent needle stick injury

[3] Cleaning and disinfection of environment and equipment.

[4] Droplet protection (mask, respinfluenza risk assessment toolors and eye shield)

[5] Proper waste mangement followed by hospitals

## Pre-hospital care:

[1] Skilled paramedical and drivers for ambulance

[2] Triple layer surgical mask use

[3] Movements should be restricted

[4] During transport, optimize vehicle ventilation (increase air exchange)

[5] Aerosol generating procedure to be avoided

[6] Disinfect the ambulance (after every shift)

## Screening center:

[1] Influenza patients should be kept in separate area

[2] Triage the patient

[3] Collect sample

[4] Provide counseling which is followed by treatment

[5] Well-ventilated area (air change)

[6] Patient seating should 1 m apart

[7] No overcrowding pf patient

[8] Facility for stretcher and wheelchair

[9] Waiting area should be cleaned and disinfected

[10] Facility of hand was and washroom *etc.*,

## Isolated ward:

[1] 10 bed facility (2000 sq. Feet area)

[2] Double door entry

[3] Changing room, nursing station

[4] Puncture proof container for sharp disposal

[5] Enough personal protective equipment kit

[6] Patient personal belonging should be minimum

[7] Keep water pitcher, cups, tissue wipe and all hygiene iteams in patient reach

[8] If air- conditioning present, ensure 12 air change/hour and filtering of exhaust air

[9] If air - conditioning not present, negative pressure could be created through 3-4 exhaust fans

[10] Visitors should be restricted

[11] Portable X-ray equipment is suggested

[12] Telephone / any other method of communication should be set up.

## Critical care:

[1] Infection control

[2] More than or equal to 12 air change

[3] Maintain negative pressure = 40 psi

[4] Would have information board outside to update relatives on clinical status

## Mortuary care:

[1] Proper hand hygiene's

[2] Cleaning of body, tidying of hair *etc.* should be done with standard precautions

The potential severity of a future H5N1 pandemic remains uncertain at this time. Recent human infections with H5N1 2.3.4.4b viruses have shown a notably lower case fatality rate compared to previous H5N1 outbreaks in Asia, during which around 50% of reported infections led to mortality [33]. As a result, the H5N1 panzootic has been marked by notable imagery of coastal regions littered with sea lion carcasses and agricultural establishments containing suffering dairy cattle that have declined after stopping their intake of food. Nonetheless, a considerable concern for researchers is the possibility of undetected transmission pathways emerging within accommodations for farm workers, swine facilities or in developing countries. The evolution of these pathways may occur without detection, attributed to restricted testing criteria, concerns regarding governmental oversight or a lack of available resources [34].

## Conclusion:

The potential for an H5N1 pandemic raises significant concerns due to its possible severe health implications for humans, rather than the notion of inevitability. It has shown its effect on the economies and nutrition of rural communities and there have been cases of zoonotic transmission that have caused serious illnesses in humans. Henceforth, failure to address this issue would result in on-going challenges for public health due to the pandemic threat associated with H5N1.

## Figures and Tables

**Table 1:** Recent world health organization report

**COUNTRY**	**2003-2009**		**2010-2014**		**2015-2019**		**2020**		**2021**		**2022**		**2023**		**2024**		**Total**	
	**Cases**	**Death**	**Cases**	**Death**	**Cases**	**Death**	**Cases**	**Death**	**Cases**	**Death**	**Cases**	**Death**	**Cases**	**Death**	**Cases**	**Death**	**Cases**	**Death**
Australia	0	0	0	0	0	0	0	0	0	0	0	0	0	0	1	0	1	0
Arzerbaijan	8	5	0	0	0	0	0	0	0	0	0	0	0	0	0	0	8	5
Bangladesh	1	0	6	1	1	0	0	0	0	0	0	0	0	0	0	0	8	1
Cambodia	9	7	47	30	0	0	0	0	0	0	0	0	6	4	10	2	72	43
Canada	0	0	1	1	0	0	0	0	0	0	0	0	0	0	0	0	1	1
Chile	0	0	0	0	0	0	0	0	0	0	0	0	1	0	0	0	1	0
China	38	25	9	5	6	1	0	0	0	0	1	1	1	0	1	0	56	32
Djibouti	1	0	0	0	0	0	0	0	0	0	0	0	0	0	0	0	1	0
Ecuador	0	0	0	0	0	0	0	0	0	0	1	0	0	0	0	0	1	0
Egypt	90	27	120	50	149	43	0	0	0	0	0	0	0	0	0	0	359	120
India	0	0	0	0	0	0	0	0	1	1	0	0	0	0	0	0	1	1
Indonesia	162	134	35	31	3	3	0	0	0	0	0	0	0	0	0	0	200	168
Iraq	3	2	0	0	0	0	0	0	0	0	0	0	0	0	0	0	3	2
Leo people's democratic republic	2	2	0	0	0	0	1	0	0	0	0	0	0	0	0	0	3	2
Myanmar	1	0	0	0	0	0	0	0	0	0	0	0	0	0	0	0	1	0
Nepal	0	0	0	0	0	1	1	0	0	0	0	0	0	0	0	0	1	1
Nigeria	1	1	0	0	0	0	0	0	0	0	0	0	0	0	0	0	1	1
Pakistan	3	1	0	0	0	0	0	0	0	0	0	0	0	0	0	0	3	1
Spain	0	0	0	0	0	0	0	0	0	2	0	0	0	0	0	0	2	0
Thailand	25	17	0	0	0	0	0	0	0	0	0	0	0	0	0	0	25	17
Turkey	12	4	0	0	0	0	0	0	0	0	0	0	0	0	0	0	12	4
United kingdom	0	0	0	0	0	0	0	0	1	0	0	0	4	0	0	0	5	0
United states of america	0	0	0	0	0	0	0	0	0	0	1	0	0	0	44	0	45	0
Vietnam	112	57	15	7	0	0	0	0	0	0	1	0	0	0	1	1	129	65
TOTAL	468	282	233	125	160	48	1	0	2	1	6	1	12	4	57	3	939	464
